# Anti-SARS-CoV-2 IgG Antibodies Post-COVID-19 or Post-Vaccination in Libyan Population: Comparison of Four Vaccines

**DOI:** 10.3390/vaccines10122002

**Published:** 2022-11-24

**Authors:** Fawzi Ebrahim, Salah Tabal, Yosra Lamami, Inas M. Alhudiri, Salah Edin El Meshri, Samira Al Dwigen, Ramadan Arfa, Asma Alboeshi, Hafsa A. Alemam, Fauzia Abuhtna, Rabeeah Altrhouni, Mohamed B. Milad, Nada A. Elgriw, Mahmoud A. Ruaua, Zakarya Abusrewil, Warda Harroush, Mwada Jallul, Fouziyah S. Ali, Farag Eltaib, Adam Elzagheid

**Affiliations:** 1 Libyan Biotechnology Research Center, Tripoli P.O. Box 30313, Libya; 2 Information Technology Department, Tripoli University, Tripoli P.O. Box 13275, Libya

**Keywords:** COVID-19 vaccines, humoral immunity, SARS-CoV-2, antibody titers, spike protein

## Abstract

Measurement of strength and durability of SARS-COV-2 antibody response is important to understand the waning dynamics of immune response to both vaccines and infection. The study aimed to evaluate the level of IgG antibodies against SARS-CoV-2 and their persistence in recovered, naïve, and vaccinated individuals. We investigated anti-spike RBD IgG antibody responses in 10,000 individuals, both following infection with SARS-CoV-2 and immunization with SARS-COV-2 AstraZeneca, Sputnik V, Sinopharm, and Sinovac. The mean levels of anti-spike IgG antibodies were higher in vaccinated participants with prior COVID-19 than in individuals without prior COVID-19. Overall, antibody titers in recovered vaccinee and naïve vaccinee persisted beyond 20 weeks. Vaccination with adenoviral–vector vaccines (AstraZeneca and Sputnik V) generates higher antibody titers than with killed virus vaccine (Sinopharm and Sinovac). Approximately two-thirds of asymptomatic unvaccinated individuals had developed virus-specific antibodies. A single dose of vaccine is likely to provide greater protection against SARS-CoV-2 infection in individuals with apparent prior SARS-CoV-2 infection, than in SARS-CoV-2-naive individuals. In addition, the high number of seropositivity among asymptomatic unvaccinated individuals showed that the number of infections are probably highly underestimated. Those vaccinated with inactivated vaccine may require more frequent boosters than those vaccinated with adenoviral vaccine. These findings are important for formulating public health vaccination strategies during COVID-19 pandemic.

## 1. Introduction

SARS-CoV-2, the virus that causes COVID-19 has infected more than 304 million people around the world as of January 9, 2022, causing more than 5.4 million deaths [1]. Herd immunity is important for resolution of herd immunity is important for resolution of pandemics [2]. Vaccines stimulate different parts of the immune system, including humoral immunity. Therefore, production of neutralizing antibodies is an indicator of vaccine efficacy. This is also observed after COVID-19 infection [3]. SARS-CoV-2 causes either symptomatic or asymptomatic infection [4] and induces the production of neutralizing antibodies in most patients [5] within two weeks after the onset of symptoms [6]. The duration of the humoral immune response and its protective efficacy are still unclear. Several studies have measured the levels of neutralizing antibodies induced by vaccination against SARS-CoV-2, which may play an important role in controlling viral infection [7]. Detection of SARS-CoV-2 antibodies indicates current or past infection [8]. However, the durability of antibody responses and their stability over time remain debatable for both natural infection and post vaccination. Some studies reported that antibodies did not decline within four months after infection [9]. Others showed rapid decline of antibody levels, their late appearance, and even seronegativity [10]. Some reports estimated that antibodies and memory cells against SARS-CoV-2 persist for at least 4–8 months [11]. Another study reported that IgG levels persist for at least nine months after exposure to the virus [12]. So far, the immunity obtained from vaccination is not lifelong or even very long-lasting, but it seems to control the infection to some extent [2]. In Libya, since the vaccine campaign began in April 2021, four types of vaccines have been administered. According to the National Centre for Disease Control in Libya, more than one million individuals have been vaccinated with the first dose and a total of 816,927 s doses have been administered [13]. However, there was a considerable delay in provision of the second dose of Sputnik V and Sinovac which caused interruption of the vaccine schedule. Evaluation of immunity after vaccination is important for determining the protection provided by the vaccine. Herein we aimed to evaluate the levels of IgG antibodies against SARS-CoV-2 and their persistence after infection and/or post-vaccination.

## 2. Material and Methods

### 2.1. Participants and Study Design

We investigated anti-spike IgG antibody responses following infection with SARS-CoV-2 and/or immunization with the first dose or with two doses of the vaccines in use in Libya. The study was conducted on the Libyan general population in several Northern cities. The study started with initial recruitment of 15,000 participants between 15 August and 31 December 2021. The participants were recruited from people visiting healthcare centers and employees in state institutions. We collected blood samples from any person >18 years old whether they had received the vaccine or not and regardless of SARS-COV-2 infection status. The specimens were obtained at multiple time points following vaccination. Exclusion criteria were pregnancy, blood or plasma transfusion during the three months preceding the study, immunosuppressive therapy, recent chemotherapy, autoimmune diseases, and renal dialysis. Blood samples of 3 mL were obtained by venipuncture using Vacutainer tubes. The samples were coded, and serum was separated and stored at −2 °C until analyzed within 48 h. A self-administered questionnaire was used to collect data on sex, age, the type of vaccines received, the date(s) of vaccination, side effects, severity of symptoms, previous COVID-19 (defined as confirmed SARS CoV-2 infection by either PCR or rapid antigen tests before vaccination) and whether the infection (if there was one) was before or after receiving the vaccine (breakthrough infection). Breakthrough infections were not counted as previous infection. Information on past medical history and influenza vaccination status were also noted. Throughout this paper the term full vaccinations indicates two vaccine doses. Partially completed questionnaires which did not contain responses for vaccination status, date of vaccination and COVID-19 infection status were excluded from further analysis. In addition, surveys which fit exclusion criteria were also removed from data analysis. The Bioethics Committee at the Biotechnology Research Center in Tripoli, Libya (Ref No. BEC-BTRC 8-2020) approved the study. The study protocol was compatible with the World Medical Association Declaration of Helsinki (Ethical Principles for Medical Research Involving Human Subjects). All participants provided written informed consent to participate. Those who agreed to participate were given an information sheet detailing the study aim, pledging anonymity of their information, and explaining that they have the right to withdraw from the study at any time.

### 2.2. Detection of SARS-CoV-2 Specific Serum Antibodies

Beckman Coulter Access Anti-SARS-CoV-2 IgG assay was used on a UniCel Dxl 600 Access Immunoassay System to detect anti-SARS-CoV-2 antibodies according to the manufacturer’s instructions (Beckman Coulter, Germany. A sample was considered reactive (positive) for anti-S IgG if the result was ≥ 10 AU/mL.

### 2.3. Statistical Analysis

A web application was developed with PHP, MySQL, and JavaScript specifically for electronically collecting survey data and initial statistical analysis. However, the statistical analysis was performed using Microsoft Excel and GraphPad Prism version 9.3. The descriptive statistics included mean, standard deviation, and percentages. Mean IgG levels were plotted at different time intervals (in weeks) between the date of serum collection and the date of vaccination. The differences between mean values were compared by the unpaired Student’s *t*-test. *p* values < 0.05 were considered statistically significant.

## 3. Results

### 3.1. Participant Selection and Characteristics of Study Group

**Figure 1:**
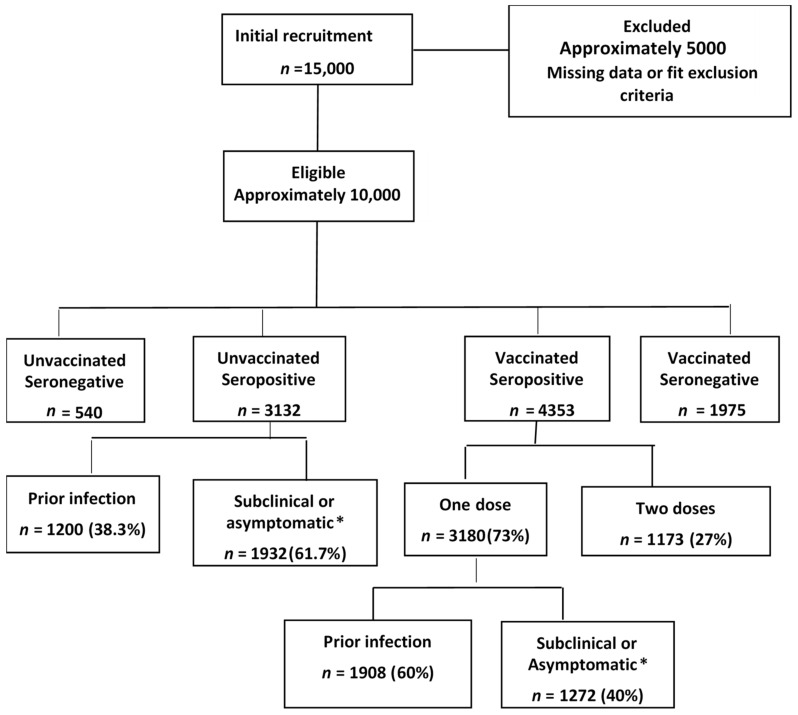
Flowchart showing recruitment and participant selection. The blood specimens were collected at multiple time points following vaccination. The second dose of the vaccine was of the same vaccine type (homologous). ***n*** indicates number and * Denotes participants who didn’t report previous infection in the survey but gave positive IgG titer indicating previous undiagnosed SARS-COV-2 infection.

Between 15 August and 31 December 2021, 10,000 adults were enrolled to the study, including both symptomatic and asymptomatic COVID-19 recoveries, as well as persons vaccinated with the first dose or with both doses of the vaccine (Figure 1).

### 3.2. Overall View of Vaccinations and Seropositivity

Of all the participants, 63.3% (6328/10,000) had been vaccinated. Among the 3132 unvaccinated individuals who were seropositive, 1932 did not report a previous infection, pointing to a rate of asymptomatic COVID-19 of 61.7% in unvaccinated individuals. The most frequently used vaccines were Sputnik V (n = 4156, 65.7%) followed by AstraZeneca (n = 1065, 16.7%) and collectively 17.5% (579/6328) of the participants had been vaccinated with either Sinopharm or Sinovac (Table 1). The overall rate of positivity was lowest for the Sinovac and Sinopharm vaccines (52.2% and 52.4%, respectively) and considerably higher for the AstraZeneca and Sputnik V vaccines (73.8% and 71.9%) (Table 1).

### 3.3. Analysis of Seropositivity by Age, Vaccination, and Prior Infection Status

The distribution of seropositivity rates by age group, apparent prior infection status, and vaccination status is shown in Table 2. Notably, all participants who had been administered one dose were seropositive, with the exception of those below 35 years of age and not previously infected, the seropositivity rate was 87.9%. An incline in positivity rate in the eldest age group was also noticed in fully vaccinated individuals regardless of infection status (Table 2).

**Figure 2:**
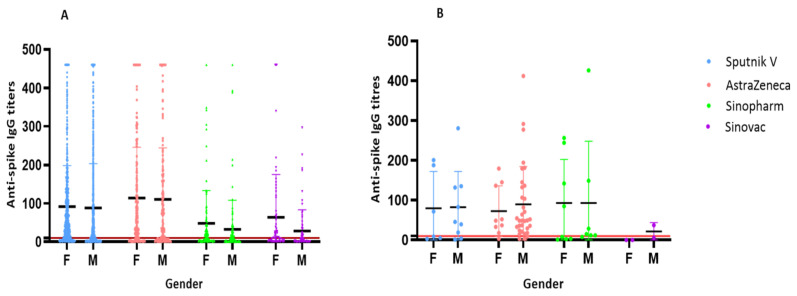
(**A**) Anti-spike IgG levels developed after each vaccine type by sex ≤ 12 weeks post- 1st vaccination dose. The IgG levels include both reactive (R: ≥10 AU/mL) and non-reactive (NR; <10 AU/mL) values. F denotes female and M denotes male. (**B**) Anti-spike IgG levels post 2nd vaccination dose. The chart shows similar median IgG titers in both male and females except in the group who received Sinopharm and Sinovac vaccines where females had significantly higher antibody response.

Males represented 54.2% of the study sample. The ages of the participants ranged from 18 to 90 years. The majority of participant were 36–45 years of age. Antibody titers against spike antigen were similar in both sexes with all vaccines except for Sinovac which showed significantly higher titers in female participants (*p* = 0.026), (Figure 2). However, the highest median titers were detected in individuals who received AstraZeneca vaccine (Figure 2). In females of >65 years who received a single dose of Sinovac with no previous infection, the mean IgG titer was 151 AU/mL compared to 48.77 AU/mL in males. Overall, the seropositivity rate was higher among unvaccinated individuals who reported prior infection (convalescent) than those who did not (asymptomatic or subclinical) (Table 2). In unvaccinated convalescent individuals, seropositivity was relatively similar in the five age groups, ranging from 77% (≤35 years) to 96% (36–45 years).

In contrast, seropositivity among unvaccinated individuals with no prior infection increased with age from 62.8% (<35 years) to 92% (55–65 years), after which there was a dip to 78% (Table 2). The seronegative unvaccinated individuals did not report previous COVID-19 in the questionnaire (Figure 1).

### 3.4. Waning Dynamics of IgG Anti-Spike RBD Levels

**Figure 3:**
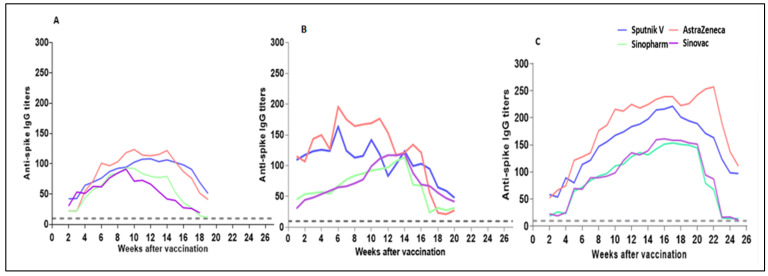
Individuals with previous SARS-COV-2 infection elicited faster and stronger antibody titers to all types of vaccinations than individuals with no previous infection (naïve). The charts shows waning dynamics in different groups according to previous infection status and vaccine dose. (**A**) Average anti-spike IgG titers after first vaccination dose in previously infected participants. (**B**) Average anti-spike IgG titers after first vaccination dose in seropositive participants who never had COVID-19 symptoms. (**C**) Trajectory of average of anti-spike IgG titers in fully vaccinated individuals (two doses). The grey dashed line in each graph indicates the threshold antibody positivity at 10 AU/mL.

From week 1 after single dose vaccination in recovered participants, the mean titers of IgG against the spike protein increased for all vaccines but were considerably higher for the AstraZeneca and Sputnik V vaccines, which, by week 11, reached 169.2 and 142.0 AU/mL, than for Sinopharm and Sinovac (92.4 and 99.3 AU/mL). However, while the levels against Sputnik V and AstraZeneca peaked on week 7 and then started a gradual decline, the IgG levels against Sinovac and Sinopharm continued to rise until week 15, when the levels against the four vaccines became very similar, ranging from 113.6 AU/mL (Sinopharm) to 123.8 AU/m (Sputnik V) (Figure 3). Thereafter, the rate of decline was more notable for AstraZeneca and Sinopharm. From week 19 to week 21, anti-Sputnik V and anti-Sinovac IgG levels were the highest. In seropositive participants without evidence of prior infection, post-vaccine antibody responses showed that positive anti-spike IgG results increased over the 3–4 weeks after the first vaccination in case of Sputnik V, AstraZeneca, and Sinovac respectively (Figure 3). In terms of Sinopharm, anti-spike IgG levels gradually started to rise with an average of 22 AU/mL at the second week and then reached a peak at the 9th week before dropping to baseline after 19th week post-vaccination.. The higher average of IgG levels was observed in Sputnik V in week 12 (108.36 AU/mL. *p* value < 0.005) while in case of AstraZeneca the maximum antibody level was seen in week 10 (132.67 AU/mL. *p* < 0.005). The higher antibody response of seropositive value were (93.7 and 91.05 AU/mL. *p* < 0.005) in Sinopharm and Sinovac respectively. In fully vaccinated respondents (two doses), the average titers of anti-spike IgG in participants who had received AstraZenca or Spunik vaccine were 257 AU/mL and 221 AU/mL, respectively (*p* value < 0.005), which is considerably higher than in those who had received only the first dose with or without prior infection (Figure 3). Similar observation were seen in Sinopharm and Sinovac as compared with patients who received the first dose (Figure 3).

## 4. Discussion

The persistence and robustness of antibody response to vaccination and infection are important parameters for assessment of vaccine efficacy and natural immune response. Public health administrations require evidence for the need of booster doses and which vaccines perform better than others. In addition, because of mostly poor compliance with protection measures, a great proportion of young population, and reduced COVID-19 testing in Libya, estimation of naturally induced immunity was important to evaluate the exact epidemiological burden of the disease. Our study sought to answer these questions through analysis of anti-spike RBD antibodies in a random population of 10,000 individuals. The study showed that nearly two-thirds of the unvaccinated seropositive had been exposed to the virus and were asymptomatic but were not previously diagnosed. This highlights that both symptomatic and asymptomatic or subclinical SARS-COV-2 infection generates good immune response. Furthermore, this prevalence in the community indicates the wide spread of COVID-19 and potential protection against reinfection, severe disease and hospitalization [14]. Administration of one vaccine dose in recovered individuals generated stronger immune response than two vaccine doses. Our results confirm previous findings that vaccinated individuals with prior infection acquire higher levels of SARS-CoV-2-specific IgG antibodies than vaccinated naïve individuals [15,16,17]. Whether these persons require a second vaccine dose is uncertain because the protective antibody level is unclear. Further studies should be performed to establish a protection threshold versus positivity threshold [18].

We also found that a single-dose of either “Sputnik V” or AstraZeneca vaccine formed a faster humoral immune response in participants who had previous infection which is similar to the findings in other studies [19,20]. IgG anti-spike antibodies were detected in less than 2 weeks in participants who received first dose with apparent prior infection with all vaccine types involved in our study. The first dose, given to individuals whose immune systems was previously stimulated by the natural infection, probably had a similar effect as the second dose in naïve individuals who received the first vaccine dose. In light of the increased demand and shortage of vaccine supply, these findings would suggest implementing a strategy of a single vaccine dose for individuals who were previously infected by SARS-CoV-2. Since the manufacturing of COVID-19 vaccines, there were questions over which vaccine performs better than the other in terms of durability and robustness. Our analysis showed that vaccination with adenoviral–vector vaccines (Sputnik V and AstraZeneca) generated higher antibody titers than for inactivated vaccines (Sinopharm and Sinovac). This finding stands in agreement with previous studies on reduced antibody titers to Sinovac and Sinopharm compared with AstraZeneca and mRNA vaccines [21,22,23]. These results suggest that giving a third booster dose would be beneficial to prevent COVID-19 infection in recipients of inactivated vaccines. These preliminary data serve as a basis for further studies on the correlation of incidence of breakthrough infections with declining neutralizing antibody or anti-spike IgG titers to guide booster shot administration. Many countries have offered booster doses to those who received Sinopharm and Sinovac regardless of the date of receiving the second dose [24]. Overall, antibody titers in recovered vaccinated individuals and naïve vaccinees persisted beyond 20 weeks for single dose recipients and beyond 24 weeks for two dose vaccine recipients. Notably, fully vaccinated respondents with or without previous COVID-19 showed high antibody levels with all vaccines but significantly higher with AstraZeneca and Sputnik V vaccines (239 AU/mL and 221 AU/mL at week 17 respectively). The decline in IgG levels over time was expected and occurred in all vaccine brands in the current study. We did not follow up persons beyond that period. This finding agreed with previously published data showing that antibodies were detected for several months after vaccination [25]. Additionally, in vaccinated individuals, IgG levels were maintained for longer periods in those who were previously infected than naïve persons [26,27]. The current study has some limitations. The study did not include children and adolescents thus missing the actual frequency of infected population in Libya. In order to identify persons with unknown previous infection in vaccinated individuals, anti-nucleocapsid antibodies should be measured which was not the case in this study. According to the frequency estimate in the unvaccinated group, we believe that a good proportion of full-dose vaccinees to have been previously asymptomatically infected. Furthermore, most fully vaccinated participants did not receive the second dose on schedule because of the delay in provision of vaccines in Libya, due to global shortage, which could reflect on interpretation of results in this group. Although this is not the normal scenario, but it is clear from the study that this delay did not affect the induction of immune response which has implications for dosing interval and vaccine administration policy.

## 5. Conclusions

SARS-CoV-2 anti-spike IgG levels among unvaccinated individuals with previous infection either symptomatic or asymptomatic showed good immune response. Vaccination in individuals with prior COVID-19 infection demonstrated higher IgG antibody levels than vaccination without prior infection. Anti-spike serum IgG following vaccination persisted beyond 20 weeks indicating probable protection against infection. Our results suggest that even greater efforts should be made to immunize more individuals either primarily or via booster doses, particularly people without prior COVID-19 infection. However, further investigations are needed to estimate the durability of these immune responses and the potential need for additional booster doses as antibody levels decline with time as well as antibody threshold for protection. Those vaccinated with inactivated vaccine may require more frequent boosters than those vaccinated with an adenoviral vaccine. These findings are important for formulating public health vaccination strategies during COVID-19 pandemic.

## Figures and Tables

**Table 1:** Distribution of vaccinated participants by sex, vaccine type, and IgG seropositivity.

Vaccine	n			Seropositive		Seronegative	
	Females	Males	Total	n	%	n	%
Sputnik V	1874	2282	4156	2988	71.9	1168	28.1
AstraZeneca	479	586	1065	786	73.8	279	26.2
Sinopharm	237	257	494	259	52.4	235	47.6
Sinovac	306	307	613	320	52.2	293	47.8
All vaccines	2896	3432	6328	4353	68.8	1975	31.2

**Table 2:** 1. Percentage positivity by age group, vaccination dose and prior infection status (*N* represents the total number of individuals in each corresponding age group). 2. *n* represents the number of seropositive individuals in each corresponding age group.

1										
	≤35 Years		36–45 Years		46–55 Years		56–65 Years		>65 Years	
	*p* = 0.0001		*p* < 0.0001		*p* = 0.0003		*p* < 0.0001		*p* = 0.0002	
	*N*	%	*N*	%	*N*	%	*N*	%	*N*	%
Vaccinated with one dose Apparent Prior infection	358	100	582	100	419	100	308	100	383	100
No prior infection	585	87.9	637	100	678	100	443	100	354	100
Vaccinated with two doses EmaApparent Prior infection	150	100	149	94.2	164	100	56	97.0	227	93.8
No prior infection	210	72.8	129	79.3	184	82.8	126	86.9	188	90.9
**2**										
	**≤35 years**		**36–45 years**		**46–55 years**		**56–65 years**		**>65 years**	
	***p*** **= 0.0001**		***p*** **< 0.0001**		***p*** **= 0.0003**		***p*** **< 0.0001**		***p*** **= 0.0002**	
	***n***	**%**	***n***	**%**	***n***	**%**	***n***	**%**	***n***	**%**
Unvaccinated EmaPrior infection	386	77.0	320	96.0	307	93.0	147	95.0	40	88.8
No prior infection	597	62.8	484	74.3	536	82.0	206	92.0	102	78.0

## Data Availability

The data are available upon reasonable request.

## References

[1] Weekly Epidemiological Update on COVID-19 - 11 January 2022. https://www.who.int/publications/m/item/weekly-epidemiological-update-on-covid-19---11-january-2022.

[2] Yoo J.-H. (2021). What We Do Know and Do Not Yet Know about COVID-19 Vaccines as of the Beginning of the Year 2021. J. Korean Med. Sci..

[3] Long Q.-X., Tang X.-J., Shi Q.-L., Li Q., Deng H.-J., Yuan J., Hu J.-L., Xu W., Zhang Y., Lv F.-J. (2020). Clinical and Immunological Assessment of Asymptomatic SARS-CoV-2 Infections. Nat. Med..

[4] Altawalah H. (2021). Antibody Responses to Natural SARS-CoV-2 Infection or after COVID-19 Vaccination. Vaccines.

[5] Iyer A.S., Jones F.K., Nodoushani A., Kelly M., Becker M., Slater D., Mills R., Teng E., Kamruzzaman M., Garcia-Beltran W.F. (2020). Persistence and decay of human antibody responses to the receptor binding domain of SARS-CoV-2 spike protein in COVID-19 patients. Sci. Immunol..

[6] Zhao J., Yuan Q., Wang H., Liu W., Liao X., Su Y., Wang X., Yuan J., Li T., Li J. (2020). Antibody responses to SARS-CoV-2 in patients with novel coronavirus disease 2019. Clin. Infect. Dis..

[7] Hodgson S.H., Mansatta K., Mallett G., Harris V., Emary K.R.W., Pollard A.J. (2021). What Defines an Efficacious COVID-19 Vaccine? A Review of the Challenges Assessing the Clinical Efficacy of Vaccines against SARS-CoV-2. Lancet. Infect. Dis..

[8] Dinnes J., Deeks J.J., Adriano A., Berhane S., Davenport C., Dittrich S., Emperador D., Takwoingi Y., Cunningham J., Beese S. (2020). Rapid, Point-of-Care antigen and molecular-based tests for diagnosis of SARS-CoV-2 infection. Cochrane Database Syst. Rev..

[9] Gudbjartsson D.F., Norddahl G.L., Melsted P., Gunnarsdottir K., Holm H., Eythorsson E., Arnthorsson A.O., Helgason D., Bjarnadottir K., Ingvarsson R.F. (2020). Humoral immune response to SARS-CoV-2 in iceland. N. Engl. J. Med..

[10] Jeyanathan M., Afkhami S., Smaill F., Miller M.S., Lichty B.D., Xing Z. (2020). Immunological considerations for COVID-19 vaccine strategies. Nat. Rev. Immunol..

[11] Dan J.M., Mateus J., Kato Y., Hastie K.M., Yu E.D., Faliti C.E., Grifoni A., Ramirez S.I., Haupt S., Frazier A. (2021). Immunological memory to SARS-CoV-2 assessed for up to 8 months after infection. Science.

[12] He Z., Ren L., Yang J., Guo L., Feng L., Ma C., Wang X., Leng Z., Tong X., Zhou W. (2021). Seroprevalence and humoral immune durability of Anti-SARS-CoV-2 antibodies in Wuhan, China: A longitudinal, population-level, cross-sectional study. Lancet.

[13] National Center for Disease Control. https://ncdc.org.ly/Ar/.

[14] León T.M., Dorabawila V., Nelson L., Lutterloh E., Bauer U.E., Backenson B. (2022). COVID-19 cases and hospitalizations by COVID-19 vaccination status and previous COVID-19 diagnosis—California and New York, May–November 2021. MMWR Recomm. Rep..

[15] Ma H., Zeng W., He H., Zhao D., Jiang D., Zhou P., Cheng L., Li Y., Ma X., Jin T. (2020). Serum IgA, IgM, and IgG Responses in COVID-19. Cell Mol. Immunol..

[16] Mantus G., Nyhoff L.E., Edara V.-V., Zarnitsyna V.I., Ciric C.R., Flowers M.W., Norwood C., Ellis M., Hussaini L., Manning K.E. (2022). Pre-Existing SARS-CoV-2 immunity influences potency, breadth, and durability of the humoral response to SARS-CoV-2 vaccination. Cell Rep. Med..

[17] Callegaro A., Borleri D., Farina C., Napolitano G., Valenti D., Rizzi M., Maggiolo F. (2021). Antibody response to SARS-CoV-2 vaccination is extremely vivacious in subjects with previous SARS-CoV-2 infection. J. Med. Virol..

[18] Wei J., Stoesser N., Matthews P.C., Ayoubkhani D., Studley R., Bell I., Bell J.I., Newton J.N., Farrar J., Diamond I. (2021). Antibody Responses to SARS-CoV-2 Vaccines in 45,965 Adults from the General Population of the United Kingdom. Nat. Microbiol..

[19] Tukhvatulin A.I., Dolzhikova I.V., Shcheblyakov D.V., Zubkova O.V., Dzharullaeva A.S., Kovyrshina A.V., Lubenets N.L., Grousova D.M., Erokhova A.S., Botikov A.G. (2021). An Open, Non-Randomised, Phase 1/2 Trial on the Safety, Tolerability, and Immunogenicity of Single-Dose Vaccine “Sputnik Light” for Prevention of Coronavirus Infection in Healthy Adults. Lancet Reg. Health-Eur..

[20] Voysey M., Clemens S.A.C., Madhi S.A., Weckx L.Y., Folegatti P.M., Aley P.K., Angus B., Baillie V.L., Barnabas S.L., Bhorat Q.E. (2021). Safety and efficacy of the ChAdOx1 NCoV-19 vaccine (AZD1222) against SARS-CoV-2: An interim analysis of four randomised controlled trials in Brazil, South Africa, and the UK. Lancet.

[21] Jeewandara C., Aberathna I.S., Pushpakumara P.D., Kamaladasa A., Guruge D., Wijesinghe A., Gunasekera B., Tanussiya S., Kuruppu H., Ranasinghe T. (2021). Persistence of Antibody and T Cell Responses to the Sinopharm/BBIBP-CorV Vaccine in Sri Lankan Individuals. medRxiv Prepr. Serv. Health Sci..

[22] Vályi-Nagy I., Matula Z., Gönczi M., Tasnády S., Bekő G., Réti M., Ajzner É., Uher F. (2021). Comparison of antibody and t Cell responses elicited by BBIBP-CorV (Sinopharm) and BNT162b2 (Pfizer-BioNTech) vaccines against SARS-CoV-2 in healthy adult humans. GeroScience.

[23] Alqassieh R., Suleiman A., Abu-Halaweh S., Santarisi A., Shatnawi O., Shdaifat L., Tarifi A., Al-Tamimi M., Al-Shudifat A.E., Alsmadi H. (2021). Pfizer-BioNTech and sinopharm: A comparative study on post-vaccination antibody titers. Vaccines.

[24] Mallapaty S. (2021). China’s COVID vaccines have been crucial—Now immunity is waning. Nature.

[25] Naaber P., Tserel L., Kangro K., Sepp E., Jürjenson V., Adamson A., Haljasmägi L., Rumm A.P., Maruste R., Kärner J. (2021). Dynamics of antibody response to BNT162b2 vaccine after six months: A longitudinal prospective study. Lancet Reg. Health-Eur..

[26] Ali H., Alahmad B., Al-Shammari A.A., Alterki A., Hammad M., Cherian P., Alkhairi I., Sindhu S., Thanaraj T.A., Mohammad A. (2021). Previous COVID-19 Infection and Antibody Levels After Vaccination. Front. Public Health.

[27] Vicenti I., Gatti F., Scaggiante R., Boccuto A., Zago D., Basso M., Dragoni F., Zazzi M., Parisi S.G. (2021). Single-dose BNT162b2 MRNA COVID-19 vaccine significantly boosts neutralizing antibody response in health care workers recovering from asymptomatic or mild natural SARS-CoV-2 infection. Int. J. Infect. Dis..

